# Effect of computerized cognitive rehabilitation in comparison between young and old age after traumatic brain injury

**DOI:** 10.1097/MD.0000000000029874

**Published:** 2022-08-19

**Authors:** Seong-Hun Kim, Dae-Won Gwak, Jae-Gyeong Jeong, Hyunwoo Jung, Yu-Sun Min, Ae-Ryoung Kim, Tae-Du Jung

**Affiliations:** a Department of Rehabilitation Medicine, Kyungpook National University Hospital, Daegu, Republic of Korea; b Department of Rehabilitation Medicine, Kyungpook National University Chilgok Hospital, Daegu, Republic of Korea; c Department of Rehabilitation Medicine, School of Medicine, Kyungpook National University, Daegu, Republic of Korea.

**Keywords:** age, cognition, cognitive dysfunction, computerized cognitive rehabilitation, traumatic brain injury

## Abstract

**Introduction:**

Cognitive dysfunction reduces patients’ quality of life and social participation with traumatic brain injury (TBI). Computerized cognitive rehabilitation is increasingly being used for cognitive therapy in TBI patients. The purpose of this study was to investigate the influence of age on the effect of computerized cognitive rehabilitation in cognitive dysfunction after TBI.

**Material and methods:**

A total of 34 patients with cognitive dysfunction after TBI were enrolled. Participants performed 30 sessions of computerized cognitive rehabilitation (Comcog) for 6 weeks. A cognitive evaluation was performed before and after treatment with Mini-Mental State Examination (MMSE) and Computerized Neurophyschologic Test (CNT).

**Results:**

There were no cognitive tests that differed between the young group and the old group at baseline. However, after computerized cognitive rehabilitation, the young group showed significant improvement compared to the old group in verbal memory, visual memory, attention, and visuo-motor coordination tests. The young group showed improvement in MMSE, verbal and visual memory, and visuo-motor coordination tests after computerized cognitive rehabilitation. In contrast, the old group showed significant improvement only in MMSE and visual learning test, one of the visual memory tests.

**Conclusion:**

Our findings demonstrate that age may be an important factor related to the effect of computer cognitive rehabilitation on cognitive dysfunction after TBI. Methodologically more ordered studies with larger sample sizes are needed in the future.

## 1. Introduction

Traumatic brain injury (TBI) is a major cause of death and disability in people under the age of 45 in the United States.^[1]^ Disorders are caused by focal and diffuse primary brain injuries due to physical damage and secondary brain injuries due to complications such as hypoxia and intracranial hypertension.^[2]^ 75% of patients with TBI have minor brain damage, 10%–12% have moderate, and 10% have severe brain damage.^[3]^ 43% of patients requiring inpatient treatment continue to have physical, cognitive, and social disabilities one year after the onset of the injury.^[4]^ Cognitive dysfunction, one of the sequelae of TBI, is a factor that contributes to disability and degrades the quality of life of people with TBI and their families.^[5,6]^ TBI causes impairments in several cognitive domains, including memory, executive function, attention, language functions, information processing speed, and visuospatial processing, and reduces independence and social participation in the academic, professional, and community settings of TBI patients.^[7–10]^

Recently, among the methods of cognitive rehabilitation, computerized cognitive rehabilitation has become an increasingly popular treatment method for people with cognitive dysfunction.^[11,12]^ Cognitive rehabilitation modifies cerebral activation and causes neuroplastic changes in patients with TBI.^[13]^ Cognitive rehabilitation was proven to be effective for cognitive and functional dysfunction, including attention, social communication, and executive function deficits in TBI patients, and has been used for treatment for cognitive dysfunction after TBI.^[14]^ Also, in a meta-analysis, it was confirmed that computerized cognitive rehabilitation is effective for visual and verbal working memory.^[15,16]^ It has been reported that computerized cognitive rehabilitation is more effective in the overall cognitive domain of TBI patients than conventional treatment.^[17]^

Among the demographic characteristics, age is an important factor in the prognosis of TBI.^[18–21]^ Hukkelhoven et al^[18]^ and Mushkudiani et al^[19]^ showed that older age is associated with mortality and poor outcomes in TBI. Rabinowitz et al.^[20]^ found that younger ages are associated with better cognitive recovery in moderate to severe TBI. In addition, Fraser et al^[21]^ reported that age predicts the recovery of cognitive function after TBI. However, studies on the effect of age on the effects of computerized cognitive rehabilitation after TBI are insufficient.

Therefore, the purpose of this study was to investigate the influence of age on the effect of computerized cognitive rehabilitation in patients with cognitive impairment after TBI.

## 2. Methods

### 2.1. Subjects

This retrospective study enrolled subjects who were diagnosed with TBI for the first time with magnetic resonance imaging and computerized tomography and transferred to the Department of Rehabilitation Medicine from March 2019 to February 2021. Inclusion criteria were: (1) Those who underwent inpatient rehabilitation therapy for cognitive dysfunction after TBI; (2) an adult 18 years or older; (3) those who could maintain a sitting position on their own when they received computerized cognitive rehabilitation for 30 minutes. Exclusion criteria were: (1) uncooperative or unable to follow instructions; (2) visual or hearing impairments that prevent computerized cognitive rehabilitation; (3) inability to sit on a chair to perform computerized cognitive rehabilitation. A total of 64 patients with TBI were recruited, and when exclusion criteria were applied, 34 patients were finally enrolled in the study. 17 people under the age of 60 were included in the young age group, and 17 people over the age of 60 were included in the old age group. The demographic characteristics (age, sex, education level, post-onset duration, and distribution of traumatic brain injury) and TBI severity (Glasgow Coma Scale [GCS] on admission) of the patients are shown in Table 1. The average age of all participants was 57.2 ± 18.1 years old, 42.8 ± 14.0 years old in the young age group, and 71.5 ± 6.7 years old in the old age group. The GCS on admission was 7.5 ± 3.6 in the young age group and 11.8 ± 3.0 in the old age group, which was lower in the young age group. The period of education was higher in the young age group, 14.4 ± 2.3 years in the young age group, and 10.6 ± 3.5 years in the old age group. Sex ratio, post-onset duration, and baseline MMSE score, distribution of traumatic brain injury did not differ between the two groups.

**Table 1:** Demographics and severity of traumatic brain injury in the participants at baseline.

	Total	Young group	Old group	*P*
n	34	17	17	
Age	57.2 ± 18.1	42.8 ± 14.0	71.5 ± 6.7	<.001*
Sex (M:F ratio)	24:10	13:4	11:6	.467
GCS on admission	9.7 ± 3.9	7.5 ± 3.6	11.8 ± 3.0	.001*
Post onset duration (d)	69.4 ± 39.1	73.9 ± 26.5	64.8 ± 49.0	.508
MMSE	18.2 ± 6.6	19.0 ± 6.3	17.4 ± 7.1	.512
Education (y)	12.5 ± 3.5	14.4 ± 2.3	10.6 ± 3.5	.001*
TBI lesion distribution				
*Intra-axial injury: **Extra-axial injury: Intra- and Extra-axial injury	10:10:14	7:5:5	3:5:9	.254
Vertical distribution [supratentorial: infratentorial]	33:1	17:0	16:1	.310
Horizontal distribution [right: left: bilateral]	10:10:14	5:6:6	5:4:8	.842

Variables are presented as mean ± standard deviation.

GCS, Glasgow Coma Scale; MMSE, Mini-Mental State Examination.

* Intra-axial injury: Diffuse axonal injury, cortical contusion, intracerebral hematoma.

** Extra-axial injury: Epidural hematoma, subdural hematoma, subarachnoid hemorrhage, intraventricular hemorrhage.

### 2.2. Computerized cognitive rehabilitation

The study participants received inpatient rehabilitation, including physical therapy and occupational therapy, and cognitive therapy for a total of 30 sessions for 6 weeks, 5 times a week through Comcog^®^ (Maxmedica, Seoul, Korea), a computerized cognitive rehabilitation program. A 30-minute treatment was performed per session. Comcog consists of 10 attention tasks to train visual and auditory attention, vigilance, dividing, and persisting attention. Comcog is a computerized cognitive rehabilitation program that is still actively used in various diseases such as stroke, TBI, mild cognitive impairment, and autism spectrum disorder.^[22–26]^

### 2.3. Cognitive assessment

The participants were evaluated before and after 6 weeks of computerized cognitive rehabilitation. As cognitive evaluation tests, Mini-Mental State Examination (MMSE) and Computerized Neuropsychologic Test (CNT, version 4.0, Maxmedica, Seoul, Korea) were performed. CNT was developed to evaluate cognitive function in patients with brain injury and consists of 6 components; verbal memory (digital span forward, digital span backward, and verbal learning), visual memory (visual span forward, visual span backward, and visual learning), attention (auditory continuous performance task (CPT), visual CPT)), visuomotor coordination (trail making A and trail making B).^[27]^ To explain the components further, the Verbal & Visual memory test is an evaluation of short-term memory, and the Digital span backward test is an evaluation of working memory as well as verbal memory.

### 2.4. Process

Before starting the cognitive rehabilitation, all participants underwent a computerized neuropsychological test (CNT) and a Mini-Mental Status Examination (MMSE) performed by occupational therapists. After the initial CNT evaluation, participants underwent 30 sessions of Comcog ® (Version 1.0, Maxmedica, Seoul, Korea) five times per week. Each session lasted for 30 minutes. The Comcog provides 10 training activities: 2 auditory processing tasks that assess response time during auditory stimulation; 2 visual processing tasks that assess response time during visual stimulation; 2 selective attention tasks that track attention in distraction; 3 working memory tasks that assess recognition and recall memory using visual, auditory, and multisensory stimulation; and 1 emotional attention task that assesses responses to pleasant or unpleasant stimulation. After 30 sessions of Computerized cognitive rehabilitation, we reevaluated CNT and MMSE to determine the cognitive function improvement. The results of each CNT component were presented based on a percentage. Then, the T-score was calculated.

### 2.5. Statistical analysis

The SPSS software (version 25 for Windows, IBM, New York, USA) was used for statistical analysis. An independent *t*-test was performed to compare the treatment effects between the young and old age groups. And paired *t*-test was performed to confirm the effect between treatments. Items that did not pass the normality test were analyzed by non-parametric tests. Results were considered statistically significant if the *P*-value was < .05.

### 2.5. Ethics approval

This study was approved by the Institutional Review Board of Kyungpook National University Chilgok Hospital (No. 2019-05-008).

## 3. Results

### 3.1. Comparison of the effects of young age group and old age group

Table 2 shows the comparison of the cognitive tests of the young age group and the old age group evaluated at baseline and after computerized cognitive rehabilitation treatment. At baseline, there were no significant differences between the two groups in any test. However, after treatment, differences were observed between the two groups in the digital span forward (young = 45.1 ± 15.7, after rehabilitation = 34.4 ± 6.9, *P* = .034) and digital span backward (young = 41.8 ± 13.1, after rehabilitation = 31.0 ± 4.8, *P* = .004) among verbal memory tests. In the visual memory tests, the young age group was better in the visual span forward (young = 39.6 ± 11.7, after rehabilitation = 30.5 ± 5.7, *P* = .003) and visual span backward (young = 39.3 ± 12.3, after rehabilitation = 30.4 ± 2.2, *P* = .009). This difference was also seen in the attention test, visual CPT (young = 51.6 ± 25.2, after rehabilitation = 30.1 ± 9.8, *P* = 0.018), and the visuo-motor coordination test, trail making B (young = 28.8 ± 21.2, after rehabilitation = 10.0 ± 14.0, *P* = .007).

**Table 2:** Comparisons of the effects of computerized cognitive rehabilitation according to age in patients with traumatic brain injury.

Assessments	Baseline			After treatment		
	Young (N=17)	Old (N=17)	*P* ^*base*^	Young (N=17)	Old (N=17)	*P* ^*after*^
MMSE	19.0 ± 6.3	17.4 ± 7.1	.563	25.1 ± 3.7	21.5 ± 5.2	.053
Verbal memory test						
Digital span forward	39.5 ± 15.1	32.8 ± 7.1	.106	45.1 ± 15.7	34.4 ± 6.9	.034*
Digital span backward	34.9 ± 12.9	30.8 ± 5.9	.518	41.8 ± 13.1	31.0 ± 4.8	.004*
Verbal learning	23.8 ± 8.9	27.1 ± 0.7	.413	28.8 ± 4.3	27.4 ± 1.6	.394
Visual memory test						
Visual span forward	34.7 ± 9.5	29.1 ± 2.6	.193	39.6 ± 11.7	30.5 ± 5.7	0.003*
Visual span backward	32.1 ± 5.5	29.7 ± 3.4	.306	39.3 ± 12.3	30.4 ± 2.2	.009*
Visual learning	35.0 ± 13.2	34.5 ± 9.3	.708	44.8 ± 9.5	41.4 ± 9.1	.131
Attention test						
Auditory CPT	28.7 ± 13.6	30.2 ± 6.0	.892	33.0 ± 17.1	27.5 ± 15.9	.099
Visual CPT	37.6 ± 23.7	33.4 ± 12.5	.734	51.6 ± 25.2	30.1 ± 9.8	.018*
Visuo-motor coordination test						
Trail making A	24.5 ± 16.0	21.0 ± 12.0	.433	31.5 ± 13.1	24.1 ± 9.4	.062
Trail making B	18.0 ± 21.5	6.3 ± 11.8	.114	28.8 ± 21.2	10.0 ± 14.0	.007*

CPT, continuous performance task, MMSE, Mini-Mental State Examination.

* *P* < .05; *P*^base^ obtained from *t*-test between young and old groups at baseline; *P*^after^ obtained from *t*-test between young and old groups after treatment.

### 3.2. Effects of computerized cognitive rehabilitation

In the young age group (Table 3), MMSE (baseline = 19.0 ± 6.3, after treatment = 25.1 ± 3.7, *P* ≤ .001) was improved after computerized cognitive rehabilitation treatment. Among the verbal memory tests, all three tests: digital span forward (baseline = 39.5 ± 15.1, after treatment = 45.1 ± 15.7, *P* = 0.026), digital span backward (baseline = 34.9 ± 12.9, after treatment = 41.8 ± 13.1, *P* = 0.030) and verbal learning (baseline = 23.8 ± 8.9, after treatment = 28.8 ± 4.3, *P* = 0.027) showed significant improvement. In the visual memory tests, superior results were observed in all visual span forward (baseline = 34.7 ± 9.5, after treatment = 39.6 ± 11.7, *P* = 0.006), visual span backward (baseline = 32.1 ± 5.5, after treatment = 39.3 ± 12.3, *P* = 0.001) and visual learning (baseline = 35.0 ± 13.2, after treatment = 44.8 ± 9.5, *P* = 0.001). In the visuo-motor coordination tests, the scores of trail making A (baseline = 24.5 ± 16.0, after treatment = 31.5 ± 13.1, *P* = 0.035) and trail making B (baseline = 18.0 ± 21.5, after treatment = 28.8 ± 21.2, *P* = 0.007) were also increased. However, no significant difference was seen in the attention test.

**Table 3:** Effect of computerized cognitive rehabilitation in young age group (n = 17).

Tests	Baseline	After treatment	*P*
MMSE	19.0 ± 6.3	25.1 ± 3.7	<.001*
Verbal memory test			
Digital span forward	39.5 ± 15.1	45.1 ± 15.7	.026*
Digital span backward	34.9 ± 12.9	41.8 ± 13.1	.030*
Verbal learning	23.8 ± 8.9	28.8 ± 4.3	.027*
Visual memory test			
Visual span forward	34.7 ± 9.5	39.6 ± 11.7	.006*
Visual span backward	32.1 ± 5.5	39.3 ± 12.3	.001*
Visual learning	35.0 ± 13.2	44.8 ± 9.5	.001*
Attention test			
Auditory CPT	28.7 ± 13.6	33.0 ± 17.1	.184
Visual CPT	37.6 ± 23.7	51.6 ± 25.2	.059
Visuo-motor coordination test			
Trail making A	24.5 ± 16.0	31.5 ± 13.1	.035*
Trail making B	18.0 ± 21.5	28.8 ± 21.2	.007*

CPT, continuous performance task, MMSE, Mini-Mental State Examination.

* *P* < .05.

In the old age group (Table 4), significant improvement was observed in MMSE (baseline = 17.4 ± 7.1, after treatment = 21.5 ± 5.2, *P* = 0.001). Among the visual memory tests, there was significant higher value in visual learning (baseline = 34.5 ± 9.3, after rehabilitation = 41.4 ± 9.1, *P* = 0.041). Verbal memory, attention and visuo-motor coordination tests showed no significant differences.

**Table 4:** Effect of computerized cognitive rehabilitation in old age group (n = 17).

Tests	Baseline	After treatment	*P*
MMSE	17.4 ± 7.1	21.5 ± 5.2	.001*
Verbal memory test			
Digital span forward	32.8 ± 7.1	34.4 ± 6.9	.220
Digital span backward	30.8 ± 5.9	31.0 ± 4.8	.905
Verbal learning	27.1 ± 0.7	27.4 ± 1.6	.655
Visual memory test			
Visual span forward	29.1 ± 2.6	30.5 ± 5.7	.288
Visual span backward	29.7 ± 3.4	30.4 ± 2.2	.281
Visual learning	34.5 ± 9.3	41.4 ± 9.1	.041*
Attention test			
Auditory CPT	30.2 ± 6.0	27.5 ± 15.9	.500
Visual CPT	33.4 ± 12.5	30.1 ± 9.8	.592
Visuo-motor coordination test			
Trail making A	21.0 ± 12.0	24.1 ± 9.4	.223
Trail making B	6.3 ± 11.8	10.0 ± 14.0	.257

CPT, continuous performance task; MMSE, Mini-Mental State Examination.

* *P* < .05.

## 4. Discussion

In our study results, there were no cognitive evaluation items that differed between the young age group and the old age group at baseline. However, after computerized cognitive rehabilitation, the young age group showed significant improvement compared to the old age group in verbal memory, visual memory, attention, and visuomotor coordination tests. The young age group showed improvement in MMSE, verbal and visual memory, and visuomotor coordination tests after 6 weeks of computerized cognitive rehabilitation. In contrast, the old age group showed significant improvement only in the MMSE and visual learning test, one of the visual memory tests.

Age is a strong predictor of overall prognosis as well as cognition in TBI patients.^[18,19,28]^ In previous studies, age is associated with short-term recovery of cognitive functions after brain injury.^[20,21,29,30]^ Griesbach et al^[31]^ showed the interactions between chronic neurophysiological changes after TBI and the neurodegenerative process caused by aging make recovery slower. Additionally, another study found that older patients show a greater decline over the first 5 years after TBI than younger patients.^[32]^ In order to plan an appropriate evaluation and rehabilitation treatment for functional maintenance and recovery of TBI patients, it is necessary to pay attention to the results that appear differently according to age as in our study. In addition, in comparison to healthy individuals in the same age range, cognitive impairments in elderly patients with TBI are evaluated in the context of normal neurocognitive aging processes. As a result, clinicians should be concerned not with the onset age of TBI, but with the difference between a normal neurocognitive aging process of an elderly patient with TBI.

We could think that differences in the effects of computerized cognitive rehabilitation according to age can be attributed to the fact that younger patients have a higher academic background and are familiar with the use of electronic devices. Computer technology is advancing rapidly and is gradually being incorporated into more and more everyday life and educational environments.^[33,34]^ Unlike the elderly, young people are much more accustomed to learning through desktop, laptop, and tablet personal computers.^[35–37]^ Another study reported that the manual and computerized versions of the Wisconsin card sorting test were not equivalent.^[38]^ Therefore, it is considered necessary to compare younger and elder patients who received only conventional cognitive rehabilitation or both conventional and computerized rehabilitation.

In 1986, Glisky et al^[39]^ reported the first computer-assisted cognitive therapy. Since then, computerized cognitive rehabilitation has become a promising treatment for various diseases such as stroke, mild cognitive impairment, dementia, and Parkinson's disease, which can cause cognitive decline in addition to TBI.^[40–43]^ Computerized cognitive rehabilitation can be performed by the patient alone and has many advantages over conventional treatment methods, such as reducing the therapist’s intervention time and providing immediate feedback on the outcome of the performance.^[44]^ With the increasing use of computerized cognitive rehabilitation, we conducted this study to investigate the age-dependent differences in its effects on TBI patients. The effect of demographic factors, including age, on computerized cognitive rehabilitation, is methodologically arranged, and large-scale research are needed in the future.

Our research has several limitations. First, only a short treatment effect of 6 weeks was observed. Cognition includes various elements of executive function, memory, visuospatial ability, and/or language; therefore, the evaluation and treatment period in this study can be a bit short to see the course of cognitive function recovery. Therefore, it was not possible to evaluate factors related to the long-term effect of more than 1 year. Second, in the young age group, the GCS on admission was worse, but the average years of education were longer than in the old age group. In addition to age, these factors may have contributed to differences in treatment effects. Third, our study could cause sampling of bias. Since only patients who needed inpatient rehabilitation after brain injury were involved, it was impossible to evaluate mild TBI without inpatient treatment. Also, we enrolled patients who could maintain a sitting position on their own when they received computerized cognitive rehabilitation for 30 minutes. Fourth, the sample size was relatively small with a total of 34 people. If the sample size is larger, the demographic characteristic may not show differences in GCS or education period between each group. Further study is needed to confirm the age-dependent effects of computerized cognitive rehabilitation in TBI.

## 5. Conclusion

Our findings demonstrate that age may be an important factor related to the effect of computer cognitive rehabilitation on cognitive dysfunction after TBI. Methodologically more ordered studies with larger sample sizes are needed in the future.

## Author contributions

Conceptualization: T.D.J.; methodology: H.W.J., T.D.J.; data curation: D.W.G., S.H.K., A.R.K., Y.S.M., T.W.N., and J.E.L.; writing—original draft preparation: D.W.G., S.H.K., H.W.J.; writing—review and editing: Y.S.M., T.D.J. All authors contributed to the article and approved the submitted version.

## References

[1] Nguyen R Fiest KM McChesney J. The international incidence of traumatic brain injury: a systematic review and meta-analysis. Can J Neurol Sci 2016;43:774–85. 27670907 10.1017/cjn.2016.290

[2] Kinoshita K. Traumatic brain injury: pathophysiology for neurocritical care. J Intensive Care. 2016;4:1–10. 27123305 10.1186/s40560-016-0138-3 PMC4847183

[3] Saatian M Ahmadpoor J Mohammadi Y. Epidemiology and pattern of traumatic brain injury in a developing country regional trauma center. Bull Emer Trauma. 2018;6:45. 10.29252/beat-060107 PMC5787363 29379809

[4] Selassie AW Zaloshnja E Langlois JA. Incidence of long-term disability following traumatic brain injury hospitalization, United States, 2003. J Head Trauma Rehabil. 2008;23:123–31. 18362766 10.1097/01.HTR.0000314531.30401.39

[5] Rabinowitz AR Levin HS. Cognitive sequelae of traumatic brain injury. Psychiatr Clin North Am. 2014;37:1. 24529420 10.1016/j.psc.2013.11.004 PMC3927143

[6] Gorgoraptis N Zaw-Linn J Feeney C. Cognitive impairment and health-related quality of life following traumatic brain injury. NeuroRehabilitation. 2019;44:321–31. 31177238 10.3233/NRE-182618

[7] Dikmen SS Corrigan JD Levin HS. Cognitive outcome following traumatic brain injury. J Head Trauma Rehabil. 2009;24:430–8. 19940676 10.1097/HTR.0b013e3181c133e9

[8] Whitnall L McMillan T Murray G. Disability in young people and adults after head injury: 5–7 year follow up of a prospective cohort study. J Neurol Neurosurg Psychiatry. 2006;77:640–5. 16614025 10.1136/jnnp.2005.078246 PMC2117429

[9] Ponsford J Kinsella G. Attentional deficits following closed-head injury. J Clin Exp Neuropsychol. 1992;14:822–38. 1474148 10.1080/01688639208402865

[10] Zaloshnja E Miller T Langlois JA. Prevalence of long-term disability from traumatic brain injury in the civilian population of the United States, 2005. J Head Trauma Rehabil. 2008;23:394–400. 19033832 10.1097/01.HTR.0000341435.52004.ac

[11] Sigmundsdottir L Longley WA Tate RL. Computerised cognitive training in acquired brain injury: a systematic review of outcomes using the International Classification of Functioning (ICF). Neuropsychol Rehabil. 2016;26:673–741. 26965034 10.1080/09602011.2016.1140657

[12] Lebowitz MS Dams-O’Connor K Cantor JB. Feasibility of computerized brain plasticity-based cognitive training after traumatic brain injury. J Rehabil Res Dev. 2012;49. 10.1682/jrrd.2011.07.0133 23516058

[13] Galetto V Sacco K. Neuroplastic changes induced by cognitive rehabilitation in traumatic brain injury: a review. Neurorehabil Neural Repair. 2017;31:800–13. 28786307 10.1177/1545968317723748

[14] Cicerone KD Goldin Y Ganci K. Evidence-based cognitive rehabilitation: systematic review of the literature from 2009 through 2014. Arch Phys Med Rehabil. 2019;100:1515–33. 30926291 10.1016/j.apmr.2019.02.011

[15] Fernández López R Antolí A. Computer-based cognitive interventions in acquired brain injury: A systematic review and meta-analysis of randomized controlled trials. PLoS One. 2020;15:e0235510. 32645046 10.1371/journal.pone.0235510 PMC7347178

[16] Fetta J Starkweather A Gill JM. Computer-based cognitive rehabilitation interventions for traumatic brain injury: a critical review of the literature. J Neurosci Nurs J Am Assoc Neurosci Nurs. 2017;49:235. 10.1097/JNN.0000000000000298 PMC5510482 28661947

[17] De Luca R Calabrò RS Gervasi G. Is computer-assisted training effective in improving rehabilitative outcomes after brain injury? A case-control hospital-based study. Disabil Health J. 2014;7:356–60. 24947578 10.1016/j.dhjo.2014.04.003

[18] Hukkelhoven CW Steyerberg EW Rampen AJ. Patient age and outcome following severe traumatic brain injury: an analysis of 5600 patients. J Neurosurg. 2003;99:666–73. 14567601 10.3171/jns.2003.99.4.0666

[19] Mushkudiani NA Engel DC Steyerberg EW. Prognostic value of demographic characteristics in traumatic brain injury: results from the IMPACT study. J Neurotrauma. 2007;24:259–69. 17375990 10.1089/neu.2006.0028

[20] Rabinowitz AR Hart T Whyte J. Neuropsychological recovery trajectories in moderate to severe traumatic brain injury: influence of patient characteristics and diffuse axonal injury–erratum. J Int Neuropsychol Soc JINS. 2018;24:237. 29032776 10.1017/S1355617717000996 PMC5957498

[21] Fraser EE Downing MG Biernacki K. Cognitive reserve and age predict cognitive recovery after mild to severe traumatic brain injury. J Neurotrauma. 2019;36:2753–61. 31017049 10.1089/neu.2019.6430

[22] Kim YH Ko MH Seo JH. Effect of computer-assisted cognitive rehabilitation program for attention training in brain injury. J Korean Acad Rehabil Med. 2003;27:830–9.

[23] Kim Y-H Yoo W-K Ko M-H. Plasticity of the attentional network after brain injury and cognitive rehabilitation. Neurorehabil Neural Repair. 2009;23:468–77. 19118131 10.1177/1545968308328728

[24] Shim J-M Kim H-H Lee Y-S. Effects of computerized neurocognitive function program induced memory and attention for patients with stroke. J Korean Phys Ther. 2007;19:25–32.

[25] Chung H-S. The Effect of Computerized Cognitive Rehabilitation Therapy (COMCOG) on children with high functioning autism spectrum disorder. J Korean Soc Neurocog Rehabil. 2018;10.

[26] Kim S Park E Cha H. Effects of cognitive training in mild cognitive impairmentmeasured by resting state functional imaging. Behav Sci. 2020;10:175. 10.3390/bs10110175 PMC7698473 33213021

[27] Kim YH Shin SH Park SH. Cognitive assessment for patient with brain injury by computerized neuropsychological test. J Korean Acad Rehabil Med. 2001;25:209–16.

[28] Dhandapani S Manju D Sharma B. Prognostic significance of age in traumatic brain injury. J Neurosci Rural Pract. 2012;3:131. 22865961 10.4103/0976-3147.98208 PMC3409980

[29] Chu B-C Millis S Arango-Lasprilla JC. Measuring recovery in new learning and memory following traumatic brain injury: a mixed-effects modeling approach. J Clin Exp Neuropsychol. 2007;29:617–25. 17691034 10.1080/13803390600878893

[30] Green RE Colella B Christensen B. Examining moderators of cognitive recovery trajectories after moderate to severe traumatic brain injury. Arch Phys Med Rehabil. 2008;89:S16–24. 19081437 10.1016/j.apmr.2008.09.551

[31] Griesbach GS Masel BE Helvie RE. The impact of traumatic brain injury on later life: effects on normal aging and neurodegenerative diseases. J Neurotrauma. 2018;35:17–24. 28920532 10.1089/neu.2017.5103

[32] de la Plata CDM Hart T Hammond FM. Impact of age on long-term recovery from traumatic brain injury. Arch Phys Med Rehabil. 2008;89:896–903. 18452739 10.1016/j.apmr.2007.12.030 PMC2600417

[33] Sutherland R Facer K Furlong R. A new environment for education? The computer in the home. Comput Educ. 2000;34:195–212.

[34] Keengwe J Onchwari G Wachira P. Computer technology integration and student learning: barriers and promise. J Sci Educ Technol. 2008;17:560–5.

[35] Couse LJ Chen DW. A tablet computer for young children? Exploring its viability for early childhood education. J Res Technol Educ. 2010;43:75–96.

[36] Kay R Lauricella S. Exploring the benefits and challenges of using laptop computers in higher education classrooms: a formative analysis. Can J Learn Technol/La revue canadienne de l’apprentissage et de la technologie. 2011;37.

[37] Chou P-N Feng S-T. Using a tablet computer application to advance high school students’ laboratory learning experiences: a focus on electrical engineering education. Sustainability. 2019;11:381.

[38] Çelik S Oğuz M Konur U. Comparison of computerized and manual versions of the Wisconsin Card Sorting Test on schizophrenia and healthy samples. Psychol Assess. 2021. 10.1037/pas0001015 33793265

[39] Glisky EL Schacter DL Tulving E. Computer learning by memory-impaired patients: acquisition and retention of complex knowledge. Neuropsychologia 1986;24:313–28. 3755511 10.1016/0028-3932(86)90017-5

[40] De Luca R Leonardi S Spadaro L. Improving cognitive function in patients with stroke: can computerized training be the future? J Stroke Cerebrovasc Dis. 2018;27:1055–60. 29221967 10.1016/j.jstrokecerebrovasdis.2017.11.008

[41] Barnes DE Yaffe K Belfor N. Computer-based cognitive training for mild cognitive impairment: results from a pilot randomized, controlled trial. Alzheimer Dis Assoc Disord. 2009;23:205. 19812460 10.1097/WAD.0b013e31819c6137 PMC2760033

[42] Lampit A Hallock H Moss R. The timecourse of global cognitive gains from supervised computer-assisted cognitive training: a randomised, active-controlled trial in elderly with multiple dementia risk factors. J Prev Alzheimers Dis. 2014;1:33–9. 29261218 10.14283/jpad.2014.18

[43] Nousia A Martzoukou M Tsouris Z. The beneficial effects of computer-based cognitive training in Parkinson's disease: a systematic review. Arch Clin Neuropsychol. 2020;35:434–47. 31942917 10.1093/arclin/acz080

[44] Bellucci DM Glaberman K Haslam N. Computer-assisted cognitive rehabilitation reduces negative symptoms in the severely mentally ill. Schizophr Res. 2003;59:225–32. 12414079 10.1016/s0920-9964(01)00402-9

